# The online interactive visual learning improves learning effectiveness and satisfaction of physicians with postgraduate year during the COVID-19 pandemic in Taiwan

**DOI:** 10.1186/s12909-023-04639-w

**Published:** 2023-09-28

**Authors:** Kung-Chen Ho, Tun-Sung Huang, Jiunn-Chang Lin, Huihua-Kenny Chiang

**Affiliations:** 1 Department of Biomedical Engineering, National Yang-Ming Chiao-Tung University, No. 155, Sec. 2, Li-Nong St, 112 Taipei, Taiwan; 2 Division of General Surgery, Department of Surgery, Mackay Memorial Hospital, 104 Taipei, Taiwan; 3 Liver Medical Center, MacKay Memorial Hospital, 104 Taipei, Taiwan; 4 Department of Medicine, MacKay Medical College, 25245 New Taipei City, Taiwan; 5 Nursing, and Management, MacKay Junior College of Medicine, 11260 New Taipei City, Taiwan

**Keywords:** Interactivity, Learning satisfaction, Online learning, Physician, Taiwan, Visual learning tools

## Abstract

**Backgrounds:**

Medical education has shifted from passive forms of teaching to more active learning strategies, particularly in response to the COVID-19 pandemic. Research has discussed the challenges and disadvantages associated with online education, but there is limited documentation on physicians’ perceptions of this sudden and unexpected transformation in medical education. This study aimed to determine the effect of online interactive visual learning on physicians’ perceptions of the effectiveness and their satisfaction with this online learning experience.

**Methods:**

We routinely recruited 64 unclassified physicians in the hospital’s postgraduate year (PGY) program between September 2021 and April 2022. PGY physicians received an online interactive visual learning course. Online (Google Form) testing and questionnaires before and after this course evaluated learning performance, learning attitude and satisfaction of these physicians.

**Results:**

The interactive online learning tools facilitated the physicians’ active learning processes by reducing their learning burden (burden vs. no burden: 4.69% vs. 68.75%) and increasing their learning interest (interest vs. no interest: 84.38% vs. 3.12%) in the online format. Post-test scores were significantly improved compared with pretest scores (post-test vs. pre-test: 5 vs. 4; p < 0.05) and their imaging recognition was markedly improved from baseline (post-test vs. pre-test: 85.19% vs. 61.11%). Levels of satisfaction correlated positively with the physicians’ learning burden (*r*_*s*_ = 0.541), learning interest (*r*_*s*_ = 0.562), and perceived benefits of imaging recognition (post-course: *r*_*s*_ = 0.508; future: *r*_*s*_ = 0.563) (all p < 0.05).

**Conclusions:**

Our online course with interactive visual learning facilitated PGY physicians’ learning performance, levels of satisfaction, interest, and perceived benefits of online learning. Hospitals and policymakers need to be aware that this learning approach can markedly enhance physicians’ academic outcomes and levels of clinical practice.

## Background

Online education is changing the way of teaching and learning. The practicality and cost-effectiveness of online education are encouraging the growth trend of transferring some aspects of education to online learning [1, 2]. Combining online learning tools with assisted active learning strategies can provide measurable benefits, such as reducing personal geographical and time constraints, providing various online platforms to disseminate materials more widely, and adapting to individual students’ learning styles [2, 3].

There have been sudden changes in curricula and academic conferences in medical institutions during the COVID-19 pandemic [4]. As the most effective way to curb the rapid spread of the virus is to maintain physical distance, the use of online teaching methods has surged worldwide [5]. Physicians’ ability to participate in these online educational activities has been compromised by a variety of factors, including a lack of structured regulations regarding optimal instructional formats, delivery times, or feedback methods, all of which have created challenges during the COVID-19 pandemic [6]. Several studies have discussed these challenges and also the disadvantages of online education during the COVID-19 pandemic [6–8]. However, there is limited investigation on physicians’ perceptions of this sudden and unexpected shift in medical education.

The limited data suggest that the medical students show a higher radiological competency after simulation teaching [9] or active learning course [10]. Additionally, the medical students reported greater levels of learning enjoyment after teaching course with interactive workshops [11]. To date, no study has elucidated the effect of interactive visual learning with an online format on the perceptions of physicians with postgraduate year (PGY) program. Therefore, the present study aimed to further determine the effect of online interactive visual learning on PGY physicians’ perceptions of the effectiveness and their satisfaction with this online learning experience.

## Methods

### Study design and participants

During COVID-19 pandemic, we routinely recruited 64 unclassified PGY physicians participating in the MacKay Memorial Hospital’s PGY program between September 2021 and April 2022, when they received online radiologic course to learn and interpret the vascular anatomy and computed tomography (CT) techniques for the abdomen. In order to assess the effectiveness of routine PGY’s program using online method during COVID-19 period, we further conducted a retrospectively study after the online education. All procedures of retrospectively study were approved by the MacKay Memorial Hospital Ethics Committee (No. 22MMHIS438e) and all participants agreed to use the coursework images and disclose relevant data for this research. All methods were carried out in accordance with relevant guidelines and regulations, and informed consent was obtained from all participants.

### Online education coursework

The online coursework was provided by the Liver Medical Center of MacKay Memorial Hospital. The instructor provided 30 min of online interactive lecture time (Google Meeting) for PGY physicians to learn the basic anatomical structure of the liver and how to use Labelme depicting software. After online interactive lecture, the PGY physicians downloaded the images from the cloud and performed depicting exercises using Labelme software. After 2 weeks of depicting exercises, the PGY physicians presented and discussed the results of their depicting exercises by 30 min of online meeting time (Google Meeting).

### Data collection

PGY physicians’ learning performance for the online education was assessed with two exams (Google Forms), before and after the study period (at 10 min before beginning the online coursework [pretest] and at 10 min after completing the online coursework [post-test]), as shown on the websites (pre-test: https://docs.google.com/forms/d/e/1FAIpQLSfGRYFS-VHTmdlasUflDn1RFLGmltr8Ebc-MkIuWkqJqy-yZw/viewform; post-test: https://docs.google.com/forms/d/e/1FAIpQLScxMH9h9JqAKarsRiWaP6RoR7MtatQpJwFWnr1NBw-3mT3iSg/viewform). The consistency of the two tests had previously been evaluated by 22 resident physicians, and yielded a significant alternate-form reliability of 0.522 (p < 0.05). Both two Google Form tests consisted of 5 items, each of which was evaluated as either 0 (insufficient answered), or 1 (adequately answered).

At 10 min after completing the online coursework, the physicians were also asked to complete an anonymous questionnaire provided by an online Google platform. The questionnaire assessed the physician’s levels of satisfaction and perceptions of the learning burden around the use of visual learning tools, their interest in imaging recognition after using the visual learning tool and the benefit of online education for future imaging recognition. Each participant was allowed to complete the questionnaire once.

Google Form questionnaires consisted of 5 parts (at 10 min of post-meeting). Respondents used the Likert scale [12] (1 = definitely ineffective, 5 = definitely effective) to evaluate their levels of satisfaction and perceived burden relating to the use of a visual learning tool, levels of interest in imaging recognition as a result of the visual learning tool, and the benefit of online education for the diagnosis of CT image after intervention and future. The study research team devised the testing and questionnaire content.

### Statistical analysis

Due to non-normal distributions of continuous variables, the Wilcoxon signed-rank test for nonparametric data and paired sample comparison was used to observe any differences between the pre- and post-test scores. Questionnaire results are expressed as percentage scores. Spearman‘s correlation analysis was performed to evaluate the strength of relations between satisfaction, burden, interest, post-class imaging recognition and future imaging recognition. A p-value of < 0.05 was considered statistically significant. All data were analyzed with SPSS software (version 25.0, 2003; SPSS Inc., Chicago, IL, USA).

## Results

### Performance of visual learning classes

**Fig. 1:**
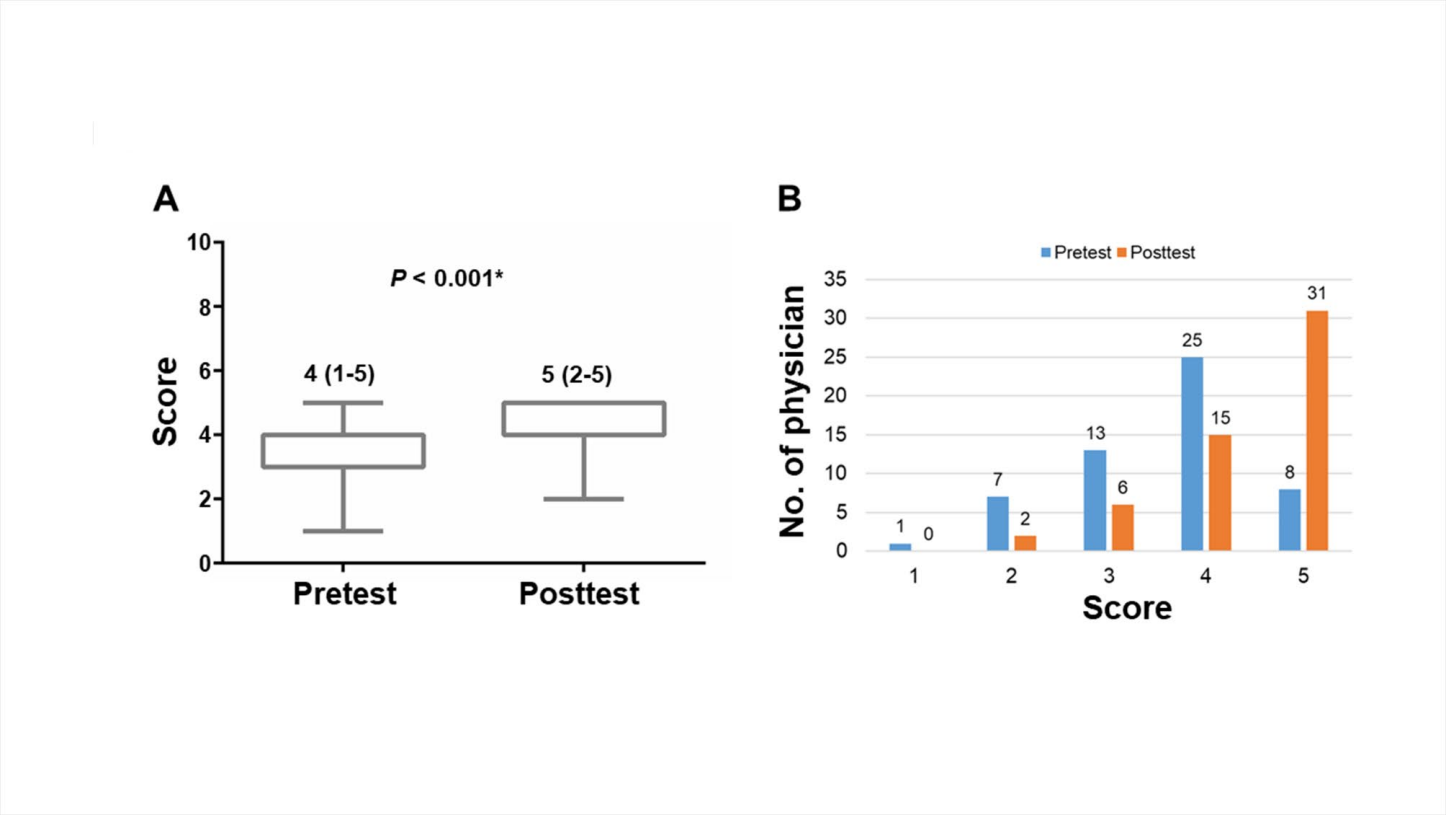
Learning performance of online course with interactive visual learning. (**A**) Pre-testing and post-testing scores. (**B**) Proportion of physicians with high performance at post-testing. *p < 0.05

In order to observe the learning development of the PGY physicians during the coursework, we evaluated learning performance by the pretest and post-test evaluations. Overall, the post-test scores were significantly higher than the pretest scores (p < 0.001, Fig. 1A), with a median of 4 (1–5) at pre-testing and a median of 5 (2–5) at post-testing. No PGY physician had a post-test score of ≤ 1. The proportion of PGY physicians with high performance (total score ≥ 4) at post-testing was at least 1.39 times higher than at pre-testing (pre-test vs. post-test: 61.11% vs. 85.19%, Fig. 1B).

### Satisfaction, burden, interest, benefit of visual learning classes

**Fig. 2:**
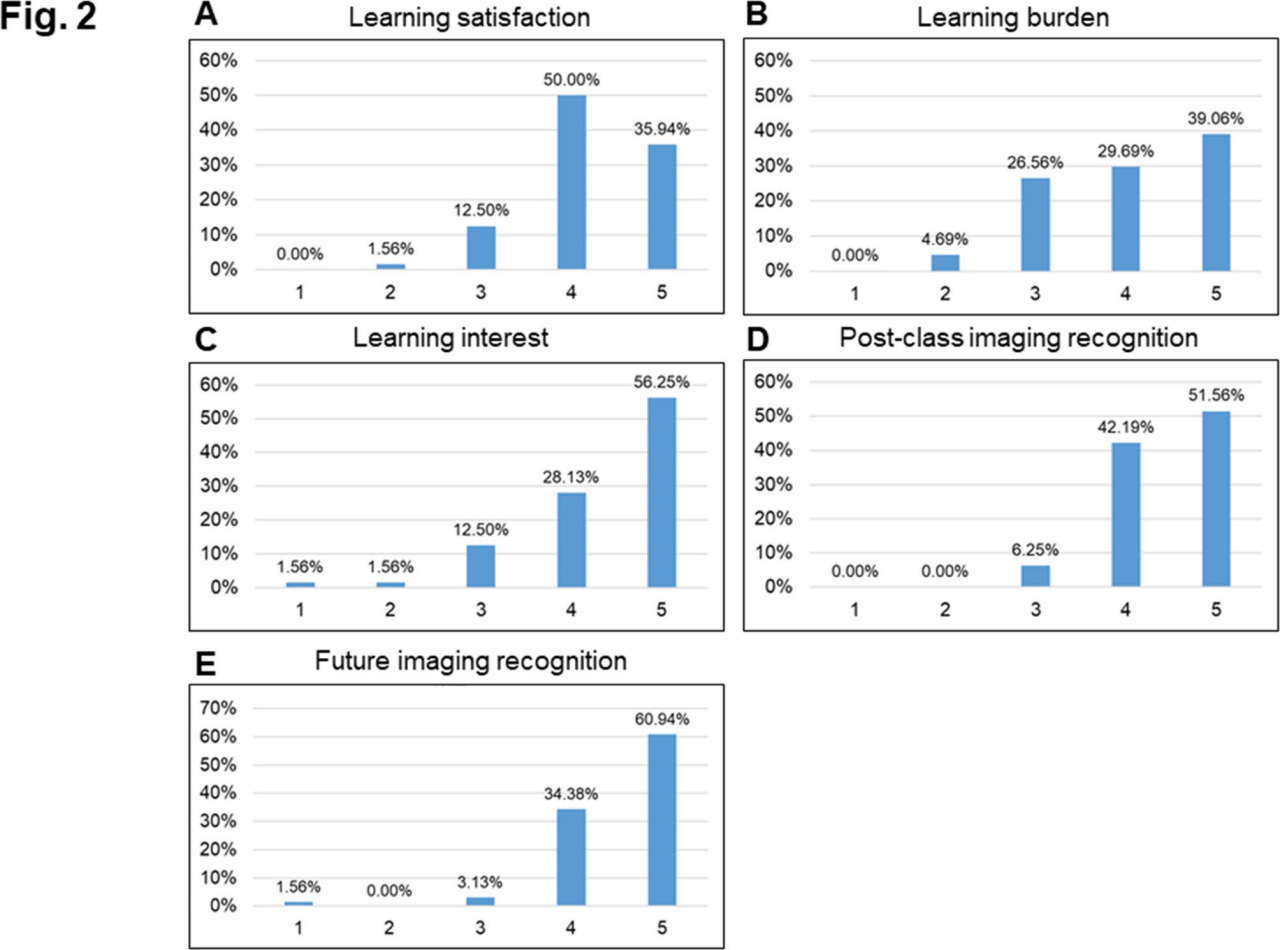
Bar diagram showing the distribution of physicians’ response to online course with interactive visual learning. (**A**) Satisfaction. (**B**) Burden. (**C**) Interest. (**D**) Post-class imaging recognition. (**E**) Future imaging recognition

Fifty-five (85.94%) PGY physicians expressed satisfaction (Likert scores ≥ 4) with the teaching methods; only 1 (1.56%) PGY physician was not satisfied (Likert score ≤ 2) (Fig. 2A). Regarding their perception of the classwork burden upon life or work, 44 (68.75%) PGY physicians had not perceived any burden (Likert scores ≥ 4); only 3 (4.69%) expressed a sense of burden (Likert scores ≤ 2) (Fig. 2B). As for the level of interest in imaging recognition due to the use of visual learning tools, 54 (84.38%) PGY physicians had an interest in imaging recognition (Likert scores ≥ 4), only 2 (3.12%) had no interest in imaging recognition (Likert scores ≤ 2) (Fig. 2C). Sixty (93.75%) PGY physicians stated that their imaging recognition had improved as a result of the classes (Likert scores ≥ 4); none stated that they had not improved in imaging recognition (no Likert scores were ≤ 2) (Fig. 2D). Sixty-one (95.32%) PGY physicians perceived that their future imaging recognition had benefited from the online classes (Likert scores ≥ 4); 1 (1.56%) PGY physician had not felt any such benefit (Likert score ≤ 2) (Fig. 2E).

**Fig. 3:**
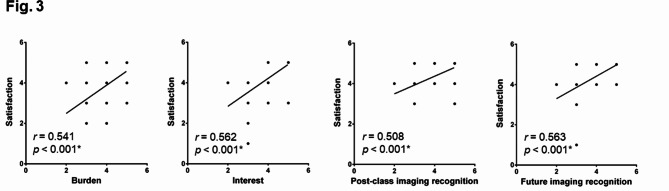
Correlation between satisfaction, burden, interest, post-class imaging recognition and future imaging recognition. Spearman’s correlation test was performed to analyze the data. *p < 0.05

To determine whether levels of satisfaction affected the continuity of online learning, we assessed correlations between satisfaction, learning burden, interest and benefits of imaging recognition. As shown in Fig. 3, Spearman’s correlation analysis revealed positive correlations between satisfaction with learning burden (*r*_*s*_ = 0.541), learning interest (*r*_*s*_ = 0.562), and benefits of imaging recognition (post-course: *r*_*s*_ = 0.508; future: *r*_*s*_ = 0.563) (p < 0.001 for all correlations).

## Discussion

This study empirically investigated the impact of interactive visual learning tools on the learning outcomes of PGY physicians, in response to the call for an empirical test of the impact of human-computer interactions on students’ online learning [13]. Based on process theory, current research highlights the potential of interactive visual learning tools for online teaching. This study has contributed to the e-learning literature by testing two important features of online learning tools, interactivity and visual learning, which promote interactive experiences, learners’ perceptions, and performance. This study provides evidence for the effects of interactivity and visual learning to PGY physicians through online courses, as well as many studies have inferred effects through correlations between learners’ self-reported perceptions and learning performance [13–15].

The results of this study provide evidence for positive correlations between PGY physicians’ interactive learning and learning performance. The previous research showing that students who have more experience in interacting with peers, instructors and educational content are more likely to have a higher level of academic self-efficacy [16]. One study has reported that students develop academic self-efficacy through observation and interaction with others [17]. Interacting with a peer’s academic performance can change a student’s academic self-efficacy by suggesting that she or he can achieve the same results [17]. Moreover, teachers can improve students’ academic self-efficacy by providing guidance and persuasive support, often acting as role models for students to successfully master the learning experience [18]. Students tend to develop their cognitive abilities and perspectives through interactions with curriculum content [19]. As they gain information and knowledge from course materials, the same interaction helps them communicate internally with themselves about teaching and learning, so that they can enhance their confidence and ability in subject knowledge [20]. According to these findings, we speculated that the positive correlations between PGY physicians’ online learning and learning performance may be due to the improvement of self-efficacy through interacting with peers, instructors and educational content, although the study did not assess self-efficacy. For this aspect, it should be further investigated in future.

This study has confirmed that interactive visual learning tools improve learning performance, as demonstrated by the higher post-test scores. Wang et al. [21] found that animation interactivity is more effective for intermediate learning (e.g., understanding concepts) than for the lowest level of learning (e.g., memory) or the highest level (e.g., advanced application). Since our study used interactive activities to help PGY physicians understand the concept of image recognition, physician participation in learning activities is understood to be at the intermediate learning level and our findings support those of Wang et al. [21]. We therefore suggest that when designing an interactive online learning activity, the content developers or instructors should consider the type of learning that physicians are expected to achieve.

Learning satisfaction represents learners’ feelings and attitudes towards the learning process, or the perceived level of achievement related to the learning desire arising from the learning experience [22]. Satisfaction has been identified as one of the most important considerations affecting the continuity of online learning [23]. Previous research into online learning has shown that learner satisfaction is an important indicator of academic achievement and the success of online learning system implementation [24]. In order to create an effective online learning environment and meet learners’ real learning needs, it is important to identify the determinants of learner satisfaction in online learning environments [25–27]. As is shown in this study, the satisfaction of online coursework is positively related to the burdens of life or work, the interest in image recognition, the improvement of image recognition after online courses, or the benefits of future image recognition. Strauß et al. [28] demonstrated that one of the reasons for the positive relationship observed between college students and online learning is the promotion of social presence. By interacting with other learners and the instructor in online learning environments, students can develop a sense of psychological contact with “real” people, even though they may be physically separated by time and distance [29]. Perception of a social presence leads to satisfaction with online learning [30].

Online lectures are the most common mode of providing radiology education. They can be delivered by the speaker as real-time (synchronous) speeches while interacting with the audience, or they can be prerecorded (asynchronous). In particular, the speaker can ask questions of the audience by using various video conferencing platforms (VCPs) such as Zoom, Skype and Google Meet, which is considered superior to the prerecorded lecture with no audience interaction. Accordingly, our study synchronized Google Meet with the online coursework to allow for real-time audience interaction with the teaching in basic anatomical structure and Labelme depicting software. After completing the depicting exercises, PGY physicians discussed their results on Google Meet. As a result of this active learning approach, the majority of PGY physicians expressed high satisfaction in online classes or interest in imaging recognition. Notably, Green et al. [31] suggest that lectures be transferred from live to online courses, while at the same time “active learning” activities (such as group work or problem sets) should be transferred into lecture sessions to increase the interest and enthusiasm of participants and improve their learning outcomes [32, 33]. Therefore, we speculated that the expressed satisfaction of PGY physicians with imaging recognition of CT scans may increase their interest in e-learning.

Ha et al. [34] report a much higher overall satisfaction of students with online activities for those who scored higher in the difficulty-level option. This finding is consistent with previous reports, in which the balance of perceived challenges and skills affects learning satisfaction only, not actual performance or perceptions of discipline learning [34, 35]. Rossin et al. [35] indicates that this may be due to the internal reward related to the task performed. Research has shown that difficulty-level questions arouse higher curiosity and interest among students, increasing their satisfaction [34]. These results imply that performed online tasks generate internal rewards (e.g., satisfaction) at first use and therefore do not require external rewards (e.g., improvements in post-test scores) to continue using the task. We therefore suggest that future online courses should contain exams or activities that incorporate the difficulty-level option, which may increase physicians’ overall satisfaction with the coursework.

Governments in many countries worldwide are working to promote the benefits of technology in the online learning process [36]. Technology saves time, enables interactive communication, improves learning efficiency, provides up-to-date learning, delivers accurate knowledge, is cost saving, facilitates flexibility around choices of location, and reduces the space and time issues associated with physical learning [37, 38]. In our study, the physicians received a total of 1 h of interactive learning (30 min of lecture and 30 min of meeting time), which significantly improved their levels of satisfaction and performance. It is obvious from these advantages that online interactive learning proved productive and beneficial for all involved (students, teachers, and support staff members) during the COVID-19 pandemic.

Several limitations of this study must be acknowledged. First, the generalizability of the study findings are limited by the fact that the study sample included PGY physicians from a single medical center in Taiwan; this sample does not represent all PGY physicians throughout Taiwan. Future studies could include more representative samples. Second, the use of self-report measures may be subject to exaggeration and lead to social desirability bias. Third, the use of a cross-sectional research design does not effectively indicate causal inferences. Thus, future research could adopt a longitudinal approach or experimental design to provide more supporting evidence about the observed relationships and their underlying mechanisms. Future studies could also test our model in different contexts, such as blended learning environments or other e-learning domains. Finally, future research testing the model developed by this study may take into account the role of technology in students’ online satisfaction.

## Conclusion

The study findings demonstrate beneficial effects of the interactive visual learning tools upon PGY physicians’ learning performance, satisfaction, interest, and benefits after enrolling in our online classes providing synchronous interactive learning. Our findings have also shed light on the underlying mechanisms that explain PGY physicians’ online learning performance and levels of satisfaction during the COVID-19 pandemic. Hospitals and policymakers need to make better decisions that ultimately influence physicians’ academic outcomes and clinical achievements.

## Data Availability

The datasets used and/or analyzed during the current study are available from the corresponding author on request.

## List of abbreviations

PGY: postgraduate year
CT: computed tomography
